# Chinese Black Truffle (*Tuber indicum*) Alters the Ectomycorrhizosphere and Endoectomycosphere Microbiome and Metabolic Profiles of the Host Tree *Quercus aliena*

**DOI:** 10.3389/fmicb.2018.02202

**Published:** 2018-09-18

**Authors:** Qiang Li, Lijuan Yan, Lei Ye, Jie Zhou, Bo Zhang, Weihong Peng, Xiaoping Zhang, Xiaolin Li

**Affiliations:** ^1^Soil and Fertilizer Institute, Sichuan Academy of Agricultural Sciences, Chengdu, China; ^2^Key Laboratory of Bio-Resource and Eco-Environment of Ministry of Education, College of Life Sciences, Sichuan University, Chengdu, China; ^3^Aquatic Geomicrobiology, Institute of Biodiversity, Friedrich Schiller University Jena, Jena, Germany

**Keywords:** truffle, ectomycorrhiza, microbial diversity, soil properties, metabolomics

## Abstract

Truffles are one group of the most famous ectomycorrhizal fungi in the world. There is little information on the ecological mechanisms of truffle ectomycorrhizal synthesis *in vitro*. In this study, we investigated the ecological effects of *Tuber indicum* – *Quercus aliena* ectomycorrhizal synthesis on microbial communities in the host plant roots and the surrounding soil using high-throughput sequencing and on the metabolic profiles of host plant roots using metabolomics approaches. We observed an increase in the diversity and richness of prokaryotic communities and a decrease in richness of fungal communities in the presence of *T. indicum*. The microbial community structures in the host roots and the surrounding soil were altered by ectomycorrhizal synthesis in the greenhouse. Bacterial genera *Pedomicrobium*, *Variibacter*, and *Woodsholea* and fungal genera *Aspergillus*, *Phaeoacremonium*, and *Pochonia* were significantly more abundant in ectomycorhizae and the ectomycorrhizosphere soil compared with the corresponding *T. indicum*-free controls (*P* < 0.05). Truffle-colonization reduced the abundance of some fungal genera surrounding the host tree, such as *Acremonium*, *Aspergillus*, and *Penicillium*. Putative prokaryotic metabolic functions and fungal functional groups (guilds) were also differentiated by ectomycorrhizal synthesis. The ectomycorrhizal synthesis had great impact on the measured soil physicochemical properties. Metabolic profiling analysis uncovered 55 named differentially abundant metabolites between the ectomycorhizae and the control roots, including 44 upregulated and 11 downregulated metabolites. Organic acids and carbohydrates were two major upregulated metabolites in ectomycorhizae, which were found formed dense interactions with other metabolites, suggesting their crucial roles in sustaining the metabolic functions in the truffle ectomycorrhization system. This study revealed the effects of truffle-colonization on the metabolites of ectomycorrhiza and illustrates an interactive network between truffles, the host plant, soil and associated microbial communities, shedding light on understanding the ecological effects of truffles.

## Introduction

Mycorrhizae are present in more than 80% of plant species, play an important role in the maintenance of the forest ecosystem and in global carbon and phosphorus cycling (Li et al., 2006; Franklin et al., 2014). Mycorrhizae can enhance nutritional values and primary productivity and modulate pest and stress resistance of the host plants (Selosse et al., 2006; Chen et al., 2017; Pérez-de-Luque et al., 2017). On the other hand, mycorrhizal fungus is supplied with carbohydrates by the host plants (Doidy et al., 2012; Ait Lahmidi et al., 2016). Mycorrhizae are commonly divided into ectomycorrhizae (ECM) and endomycorrhizae (Allen et al., 2003). It is known that colonization of plant tissues by mycorrhizal fungus results in an increase of sucrose catabolism, an increase of some organic acids, and a decrease of some amino acids (Laparre et al., 2014; Saia et al., 2015). In spite of the importance of ECM associations for plant vitality, we have little information on the molecular and physiological mechanisms of ectomycorrhizal synthesis (Larsen et al., 2011; Tschaplinski et al., 2014; Martin et al., 2016). Most of our current knowledge are based on the studies of plants that interact with arbuscular mycorrhizae (AM), which may differ in many respects of those that interact with EM fungi (Luo et al., 2009; Vayssieres et al., 2015; Watts-Williams and Cavagnaro, 2015).

In the post-genomic era of systems biology, metabolomics approaches gain their popularity to assess the metabolomes of organisms in a comprehensive and unbiased way and to uncover unknown regulation patterns (Rasmussen et al., 2012). However, most knowledge on the modulation from diverse metabolites are dependent on the studies of a few model plant species and it is difficult to transfer the findings from these models to other plant systems (Schweiger et al., 2014). Besides, targeted analyses which focus on the analysis of a few well-characterized metabolites fail to complete the full metabolic picture of plant responses (Schweiger et al., 2014; Schweiger and Muller, 2015). Metabolic profiling is expected to enhance the insight into the ECM symbiotic interactions with adequate resolution (Vayssieres et al., 2015). Being downstream of the transcriptome and proteome, the metabolome is closest to diverse biological functions and thus decisive for ecological interactions between the host plants and the mycorrhizal fungi, for example, truffles (de Vries et al., 2011).

Truffles, belonging to the *Tuber* genus (Ascomycota, Pezizales), are a group of fungi that produce hypogeous fruiting bodies (Poma et al., 2006; Benucci and Bonito, 2016). Their fruiting bodies are considered very precious and delicious food because of their unique fragrance (Pacioni et al., 2014; Schmidberger and Schieberle, 2017). *Tuber* spp. is an ectomycorrhizal fungus, which does not produce fruiting bodies without symbiotic association with its host plants. Truffles can form a symbiotic relationship with several genera of trees, including *Abies*, *Corylus*, *Pinus*, and *Quercus* (Zitouni-Haouar et al., 2014; Marozzi et al., 2017). The black truffle (*Tuber melanosporum*) and the white truffle (*T. magnatum*) have been highly valued in the European market because of their unique flavors (Vita et al., 2015; Payen et al., 2016). *Tuber indicum*, commonly known as the Chinese black truffle, is the main commercial truffle species in China (Garcia-Montero et al., 2008). It shows a close phylogenetic relationship with *T. melanosporum* and has similar morphological features (Chen et al., 2011). Ribonuclease and polysaccharides isolated from their fruiting bodies or fermentation systems showed strong antitumor, antioxidant, and antiproliferative activities (Xiao et al., 2014; Zhao et al., 2014). Due to the decreased yield of wild truffles, the synthesis and artificial cultivation of these ectomycorrhizae have attracted more and more attention. At present, *T. indicum* ectomycorrhizae have been successfully synthesized with Chinese indigenous plants, such as *Pinus armandii* and *Castanea mollissima* (Geng et al., 2009). The *P. armandii* and *Quercus aliena* mixed forest is one of the major forest types in many areas of China (Yu et al., 2014). *Q. aliena* is widely distributed in China and has a strong environmental adaptability. Its roots are well-developed and colonized by various mycorrhizae. *T. indicum* was successfully cultivated in artificial truffle orchards with *Q. aliena* as the host tree in China. However, the plant’s metabolic components in response to the ectomycorrhizal synthesis, especially to truffle ectomycorrhizal synthesis, remain unknown, which limits our understanding of the interactive relationships between this important ectomycorrhizal fungus (*T. indicum*) and its host plants (Splivallo et al., 2009).

As one of the most important members of the ectomycorrhizal fungi, a truffle plays an important role in the terrestrial ecosystems. Some black truffles often form brûlé (an area devoid of herbaceous cover) around the host trees, affecting the biodiversity of bacteria and fungi in the brûlé area (Mello et al., 2013, 2015; De Miguel et al., 2014). Being dominant, *Tuber* spp. is predicted to affect the associated soil fungal communities (Napoli et al., 2010; Streiblova et al., 2012), plausibly leading to the formation of the brûlé region. Different truffle species were found to exert different competitive effects on other mycobionts (Garcia-Barreda and Reyna, 2012; Taschen et al., 2015). ECM can be beneficial to plant productivity by enhancing plant growth or resistance to abiotic stress (Alvarez-Lafuente et al., 2018). They also can improve water and nitrogen acquisition of the host plants and play a key role in the nutrition acquisition of forest trees (Liese et al., 2018). The colonization of ECM induces higher soil porosities, which has been proven to play a crucial role in achieving success in black truffle plantations (Alonso Ponce et al., 2014). Our previous studies have shown that the growth of *T. indicum* alters the microbial communities of ectomycorrhizosphere soil and ectomycorrhizae of *P. armandii* (Li et al., 2017).

However, the effects of the truffle ectomycorrhizal symbiosis on soil microbial communities and the endophytes of *Q. aliena* have not been investigated. The differences or the common effects of truffle colonization on microbial communities associated with different host species remain unknown. Hence, we conducted high-throughput sequencing to access the impact of truffle ectomycorrhizal synthesis on microbial communities and to identify the core microbial flora in the rhizosphere soil and the *Q. aliena* roots. In the present study, we also determined the metabolic responses of the host plant (*Q. aliena*) to the colonization of the *T. indicum* using function prediction tools and metabolomics approaches. To our knowledge, this is the first time the metabolomics was used to reveal the metabolisms of truffles in response to truffle ectomycorrhization, with the aims to enhance the insight into the biological and chemical interactions between the host plant and *T. indicum* and to produce practical guides on the commercial cultivation of truffles sustainably.

## Materials and Methods

### Ectomycorrhizal Synthesis and Sampling Strategy

**FIGURE 1:**
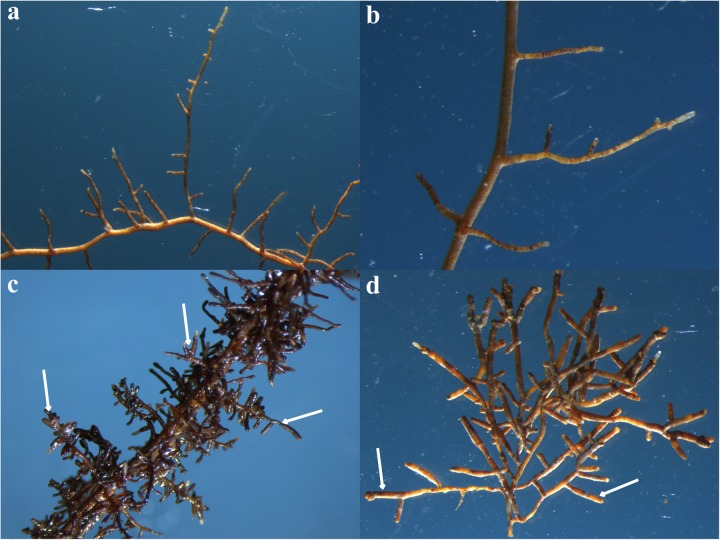
Root tips of *Quercus aliena* without *Tuber indicum* **(a,b)** or with *T. indicum* partner **(c,d)**. The white arrows indicate the inflated root tips which are colonized by *T. indicum*. These pictures were photographed using routine stereo microscopes (M80, Leica Microsystems, Inc., Buffalo Grove, IL, United States) under different magnification. **(a)** 10; **(b)** 20; **(c)** 13; **(d)** 16.

Ectomycorrhizae of *Q. aliena* with *T. indicum* were synthesized in a greenhouse in Chengdu, Sichuan, China, according to our previous described methods (Li et al., 2017) with some modifications. Briefly, the *Q. aliena* seeds were first surface sterilized with 30% H_2_O_2_ for 4 h and washed with distilled water for three times. Substrate I (consisting of vermiculite, perlite, and water at a ratio of 1:1:1, v/v/v) and Substrate II (consisting of peat, vermiculite, organic soil, and water at a ratio of 1:1:1:0.9, v/v/v/v) were autoclave sterilized for 90 min at 121°C prior to use. The surface-sterilized seeds were first sown in a plastic container filled with sterilized Substrate I to germinate. A month later, the *Q. aliena* seedlings were transplanted into separate plots filled with 1 L of sterilized Substrate II. The final pH of the homogenized substrates was adjusted to 7.5 by adding calcium hydroxide. The spore powder of *T. indicum* was obtained by blending ascocarps, surface sterilized with 75% alcohol and soaked with sterile water, to incite the spores to be released and to germinate. Ascocarps were collected from a mountain, on which *T. indicum* formed a symbiotic relationship with pine trees. We inoculated 2 g of the *T. indicum* spore powder into the substrate of each *Q. aliena* seedling. Six *Q. aliena* seedlings that were not inoculated with the truffle spores served as controls. Each treatment had six biological replicates. All the 12 pots were maintained in the greenhouse under the same conditions. Plants were watered every 3 days. After 5 months, the ectomycorrhizae were successfully obtained in all the six seedlings inoculated with truffle spores; the other six control seedlings were not colonized by truffles, as revealed by morphology analysis (**Figure 1**). The mycorrhization was checked using a stereomicroscope and the colonization rate was about 49% according to the method described by Andres-Alpuente et al. (2014). The identification of the *T. indicum* mycorrhizae was checked by ITS-rDNA sequence analyses according to Geng et al. (2009). *Q. aliena* root tips (approximately 0.5 g), mycorrhized with truffle mycelia (ECM) or without (CK), were surface sterilized and preserved at -80°C, prior to metabolomics analysis and MiSeq sequencing. The root-surrounding (rhizosphere) soil (about 100 g) was also collected and immediately subjected to chemical determination and microbial diversity analysis. The soil surrounding the root tips of *Q. aliena* mycorrhized with *T. indicum* was assigned to ECM.S. The soil surrounding the roots of the control trees without *T. indicum* partner was assigned to CK.S in the following text. Four replicates were used for microbiome analysis whereas six for metabolic analysis in order to reduce the error in metabolome detection.

### Determination of Soil Properties

The properties of soil samples surrounding the roots were analyzed according to our previously described method (Li et al., 2016, 2017). Briefly, soil pH was measured by dissolving air-dried soil in distilled water at the ratio of 1:5. Organic matter content was detected using the Tyurin method (Itoh et al., 2013). Total nitrogen was determined by the Kjeldahl method (Itoh et al., 2013). Total phosphorus was measure using molybdenum antimony ascorbic acid spectrophotography. Total potassium was determined by flame photometry. Available nitrogen was quantified using alkali solution diffusion method. Available phosphorus was determined using baking soda leaching – molybdenum antimony colorimetric method. Available potassium was determined by ammonium acetate extraction – flame photometry. Soil magnesium and calcium were measured by inductively coupled plasma optical emission spectroscopy (Optima 2000 DV, PerkinElmer, United States), with yttrium as the internal standard. All the experiments were conducted in triplicates.

### DNA Extraction and MiSeq Sequencing

The ectomycorrhizae or the control roots of *Q. aliena* were first surface sterilized according to our previous described method (Li et al., 2017). Total genomic DNA of the surface sterilized roots were extracted using cetyltrimethyl ammonium bromide (CTAB) method (Clarke, 2009). The genomic DNA of the root-surrounding soil was extracted using the Soil DNA Kit (D5625-01, Omega Bio-tek, Inc., Norcross, GA, United States) following the manufacturer’s instructions. DNA quality was monitored on 1% agarose gels. The DNA per sample was diluted to 1 ng/μL using sterile water. The partial 16S rRNA gene (V4 region) was amplified using the universal primers 341F-806R and the fungal partial ITS gene (ITS1 region) using primers ITS1F – ITS2, with barcodes added as markers to distinguish the individual samples (Huang et al., 2015; Fu et al., 2016). To study the endophytic and soil-borne microbial communities, Illumina’s MiSeq sequencing was used. The MiSeq sequencing libraries were constructed according to our previous described methods (Li et al., 2017). Libraries were sequenced on an Illumina MiSeq platform and 250 bp paired-end reads were generated (Caporaso et al., 2012).

### MiSeq Sequencing Data Analysis

The paired-end raw reads were first filtered using a series of quality control methods, including removing the barcode and primer sequences, merging overlapped sequences and removing chimera sequences, to obtain high quality data (Haas et al., 2011; Magoc and Salzberg, 2011; Bokulich et al., 2013). Sequence analysis was performed using the Uparse software (Edgar, 2013). The operational taxonomic units (OTUs) were assigned at ≥97% similarity threshold. One representative sequence per OTU was screened for further taxonomic annotation. Bacterial and fungal taxonomic annotation was performed using RDP 3 classifier (Wang et al., 2007), based on the Greengenes database (DeSantis et al., 2006) and the UNITE database (Koljalg et al., 2005), respectively. To minimize the difference in sequencing effort across samples, the abundance of each OTU per sample was normalized by rarefying the OTUs to the smallest sample library size. Subsequent analyses of alpha diversity and beta diversity were performed based on the normalized OTU data using QIIME Version 1.7.0 (Caporaso et al., 2010). Alpha diversity was applied to analyze the species complexity of each sample with five ecological indices, including observed species, Chao1 (Chao, 1984), ACE (Chao and Lee, 1992), Shannon, and Simpson. The metabolic functions of the bacterial communities were predicted using Phylogenetic Investigation of Communities by Reconstruction of Unobserved States (PICRUSt) software (Langille et al., 2013) based on the classification of the microbial metabolic functions present in the KEGG database and the COG database. The fungal functional group (guild) of the OTUs was inferred using FUNGuild v1.0 (Nguyen et al., 2016).

Data of the above microbial community analysis are presented as means ± standard deviations (SD) of four biological triplicates for each treatment. Statistical analysis was carried out by one-way analysis of variance (ANOVA) using SPSS 19.0. Least significant difference (LSD) was performed to test if the ANOVA result between the treatment groups was significant at *P* < 0.05. Spearman correlation coefficient (rho) between soil properties and indicators of bacterial communities were calculated using SPSS 19.0.

### Metabolite Extraction

The frozen plant root tips (100 mg) were ground into fine powders under liquid nitrogen using a high flux organization grinding apparatus (JQ-M4, JIQUN, Co., Ltd., Hebei, China). Metabolite extraction for ultra-high performance liquid chromatography time of flight tandem mass spectrometry (UHPLC-TOF-MS/MS) was carried out following the previously described method (Weckwerth et al., 2004; Rao et al., 2016). Briefly, 1000 μL of methanol (pre-cooled at -20°C) was added to the fine powders, followed by vortex-mixing for 30 s. The mixture was transferred into an ultrasound machine at room temperature for 30 min. Then 750 μL of chloroform (pre-cooled at -20°C) and 800 μL of deionized water (dH_2_O, 4°C) were added. The mixture was vortex-mixed for 1 min and centrifuged for 10 min (12000 rpm). The supernatant was transferred into a new Eppendorf tube and passed through a 0.22 μm membrane filter. In order to avoid system errors, all of the samples were injected into the apparatus randomly. We also introduced the quality control (QC) samples to ensure the stability of the system.

### Detection of Metabolites by UHPLC-TOF-MS/MS

Acquity Ultra Performance LC system (Waters, United States) was used in this study and operated according to the instructions. Acquity UPLC BEH C18 column (2.1 mm × 100 mm, 1.7 mm, Waters, United States) was maintained at 40°C. A sample volume of 4 μL with the partial loop injection mode was used in all of the experiments. The temperature of the autosampler was kept at 4°C. Gradient elution of the analytes was carried out with 0.1% formic acid in water (A) and 0.1% formic acid in acetonitrile (B) at a flow rate of 0.25 mL/min. An increasing linear gradient of solvent B (v/v) was used as follows: 0∼1 min, 2% B; 1∼9.5 min, 2%∼50% B; 9.5∼14 min, 50%∼98% B; 14∼15 min, 98% B; 15∼15.5 min, 98%∼2% B; 15.5∼17 min, 2%. The chromatographic column was kept balanced for 1 min prior to taking the next sample. The leucine enkephalin was used as the lock and spray (0.4 ng L^-1^, 0.1% formic acid, CAN/H_2_O 50/50).

The ESI-MS experiments were executed on the Thermo LTQ-Orbitrap XL mass spectrometer with the spray voltage of 4.8 and -4.5 kV in positive and negative modes, respectively. Sheath gas and auxiliary gas were set at 45 and 15 arbitrary units, respectively. The capillary temperature was 325°C. The voltages of capillary and tube were 35 and 50 V, -15 and -50 V in positive and negative modes, respectively. The Orbitrap analyzer scanned over a mass range of m/z 89–1,000 for full scan at a mass resolution of 60,000. Data dependent acquisition (DDA) MS/MS experiments were performed with CID scan. The normalized collision energy was 30 eV. Dynamic exclusion was implemented with a repeat count of 2 and an exclusion duration of 15 s.

### Metabolic Profiling Analysis

Metabolite peaks in the LC–MS/MS data were extracted by Waters Masslynx 4.1 software. Raw MS files were turned into mzXML format by Proteowizard software (v3.0.8789) and sequentially processed by XCMS software running under R (v3.1.3) for chromatographic matching, metabolic features detection, and aligning all metabolite peaks of the LC–MS data. In the end, all of the peak areas were normalized by the sum and then subjected to statistical analyses.

Multivariate analyses were performed using Soft Independent Modeling of Class Analogy (SIMCA)-P (version 11.0, Umetrics AB, Umeå, Sweden). All variables were UV (Unit Variance) scaled before partial least squares-discriminant analysis (PLS-DA) and principal component analysis (PCA). A loading plot was used to show the contributions of different variables to the samples. The notable different metabolites were screened by the loading plot in PLS-DA and PCA. Subsequently, independent *t-*test was performed to determine significant metabolites between different groups (*P* ≤ 0.05). Hierarchical clustering analysis (HCA) was carried out using R software (v3.1.3) to group and visualize metabolite profiles. Metabolic pathways were mapped according to pathway topology analysis on Metaboanalyst and KEGG metabolic database^1^.

Pearson correlation coefficient was used to analyze the correlation between the metabolites. Data with r^2^ ≥ 0.49 and false discovery rate (FDR) ≤ 0.05 were screened for the final link drawing. Correlation coefficient was calculated by R language. The Cytoscape 3.02^2^ was used for metabolic pathway drawing and visualization.

## Results

### Prokaryotic Alpha Diversity Indices

High-throughput sequencing technique was used to detect the differences in microbial diversity in the *Q. aliena* root tips and the surrounding soil with and without the colonization of *T. indicum*. The rarefaction curves of the prokaryotic (bacterial and archaeal) and fungal OTUs in different samples are shown in **Supplementary Figure S1**.

An average of 57,963 reads were obtained per sample after quality control procedures. Altogether, 42 phyla, 165 classes, and 930 genera of bacteria and archaea were classified in all samples. The number of OTUs assigned at the 97% similarity threshold ranged from 976 to 2067 across samples. The analysis of the estimated richness indices (e.g., observed species, Chao1 and ACE) revealed that the ectomycorrhizosphere soil (ECM.S) and the roots without *T. indicum* partners (CK) harbored the highest and the lowest prokaryotic richness, respectively (**Table 1**). The two diversity indices (e.g., Shannon and Simpson) indicated that prokaryotic diversity was highest in the ectomycorrhizosphere soil (ECM.S) and the rhizosphere soil without *T. indicum* partner (CK.S) and lowest in the roots without *T. indicum* partners (CK).

**Table 1:** Community richness and diversity indices of bacteria and fungi in the roots and rhizosphere soil of *Quercus aliena* with or without *Tuber indicum* partner.

Sample	CK	ECM	CK.S	ECM.S
**Bacterial indices**	Observed species	1136 ± 109c	1722 ± 56b	1737 ± 57b	1983 ± 90a
	Chao1	1027 ± 249c	1355 ± 100c	1776 ± 130b	2276 ± 107a
	ACE	1081 ± 266d	1432 ± 103c	1796 ± 168b	2346 ± 128a
	Shannon	5.28 ± 0.63c	6.53 ± 0.28b	9.17 ± 0.04a	9.27 ± 0.07a
	Simpson	0.84 ± 0.02c	0.90 ± 0.02b	0.99 ± 0.00a	1.00 ± 0.00a
**Fungal indices**	Observed species	126 ± 24c	97 ± 25c	352 ± 26a	256 ± 34b
	Chao1	83 ± 23c	52 ± 13c	298 ± 27a	215 ± 31b
	ACE	83 ± 22c	52 ± 13c	301 ± 31a	215 ± 31b
	Shannon	1.94 ± 0.76b	1.03 ± 0.14b	4.34 ± 0.56a	3.35 ± 0.66a
	Simpson	0.60 ± 0.22b	0.39 ± 0.08c	0.85 ± 0.09a	0.78 ± 0.08ab


*ECM, ectomycorrhizae; ECM.S, ectomycorrhizosphere soil; CK, roots of *Quercus aliena* without *T. indicum* partner; CK.S, rhizosphere soil of *Quercus aliena* without *T. indicum* partner. Each mean ( ± SD) was calculated from triplicates of the same treatment group. The values followed by different lowercase letters indicate significant differences (*P* < 0.05) between the treatment groups in a row.*

### Fungal Alpha Diversity Indices

The rhizosphere soil without *T. indicum* partner (CK.S) exhibited the highest fungal richness, whereas the root samples with *T. indicum* partner (ECM) showed the lowest fungal richness (**Table 1**). The fungal diversity indices (e.g., Shannon and Simpson) were significantly higher in soil (CK.S and ECM.S) than in the roots (CK and ECM), irrespective of *T. indicum* inoculation.

### Taxonomic Analyses of Prokaryotic Communities

**FIGURE 2:**
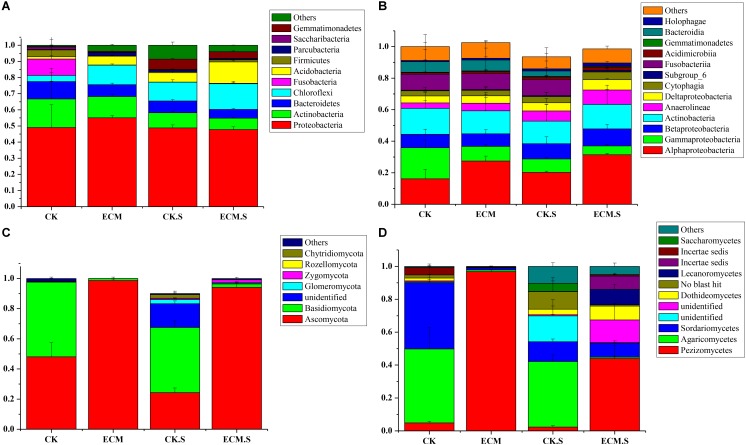
Taxonomic composition of bacterial and fungal communities associated with *Q. aliena* root tips and the surrounding soil at the phylum and class levels analyzed using barcoded sequencing. ECM, ectomycorrhizae; ECM.S, ectomycorrhizosphere soil; CK, roots of *Q. aliena* without *T. indicum* partner; CK.S, rhizosphere soil of *Q. aliena* without *T. indicum* partner. **(A)** Bacterial phyla; **(B)** bacterial classes; **(C)** fungal phyla; **(D)** fungal classes. The Y axis indicated the relative abundance of each taxon. All experiments were conducted in triplicates.

At the phylum level, 25 prokaryotic phyla were shared across all the samples. *Proteobacteria*, *Actinobacteria*, *Bacteroidetes*, and *Chloroflexi* were the dominant bacterial phyla in all samples (**Figure 2A**). The relative abundance of *Actinobacteria* and *Chloroflexi* were significantly higher in ECM and ECM.S samples than the corresponding controls (*P* < 0.05). The archaeal phylum *Euryarchaeota* was only detected in CK.S, accounting for less than 0.1% of the prokaryotic community (data not shown).

At the class level, *Alphaproteobacteria*, *Gammaproteobacteria*, *Actinobacteria*, *Betaproteobacteria*, and *Anaerolineae* were dominant (**Figure 2B**). *Alphaproteobacteria* was more abundant in ECM than in CK, whereas *Gammaproteobacteria* was more abundant in CK than in ECM (*P* < 0.05). *Alphaproteobacteria* was more abundant in ECM.S than in CK.S, whereas *Gammaproteobacteri*a was more abundant in CK.S than in ECM.S (*P* < 0.05).

**FIGURE 3:**
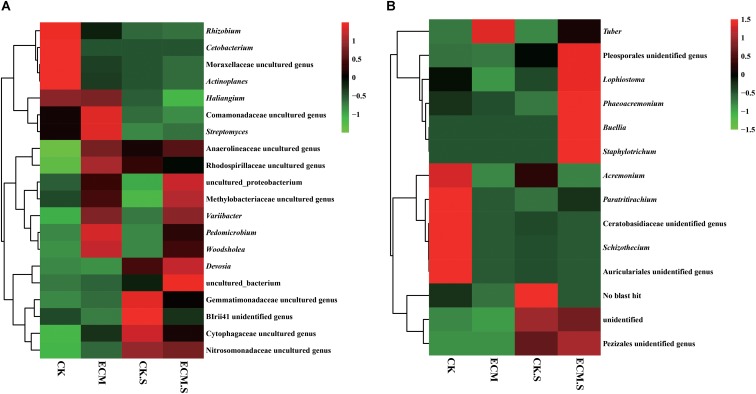
Heat-map analysis of the most abundant (average relative abundance > 0.01) bacterial **(A)** and fungal **(B)** genera in *Q. aliena* roots and the surrounding soil. ECM, ectomycorrhiza; ECM.S, ectomycorrhizosphere soil; CK, roots of *Q. aliena* without *T. indicum* partner; CK.S, rhizosphere soil of *Q. aliena* without *T. indicum* partner. The relative abundance of the samples at genus level increased with the increase of the color block value.

At the genus level (**Supplementary Figure S2**), *Actinoplanes* (average 2.35%), *Cetobacterium* (1.88%), *Pedomicrobium* (1.59%), *Devosia* (1.49%), *Variibacter* (1.40%), *Woodsholea* (1.29%), and *Streptomyces* (1.25%) were the dominant genera in all samples (**Figure 3A**). Out of the 25 most abundant prokaryotic genera, 20 were significantly more abundant in ECM than in CK, such as *Aquicella*, *Arthrobacter*, *Bacillus*, *Chlorothrix*, *Solibacter*, *Geobacillus*, *Hyphomicrobium*, *Humatobacter*, *Pedomicrobium*, *Rhodomicrobium*, *Sandaracinus*, *Sporecytophaga*, *Streptomyces*, *Sulfuritalea*, *Terrimonas*, *Turneriella*, and *Woodsholea* (*P* < 0.05, **Supplementary Figure S3A**). The prokaryotic genera such as marine group, *Mesorhizobium*, *Pseudolabrys*, *Variibacter*, and *Woodsholea* were significantly more abundant in ECM.S than in CK.S (*P* < 0.05, **Supplementary Figure S3B**).

### Taxonomic Analyses of Fungal Communities

At the phylum level, *Ascomycota* and *Basidiomycota* were the two dominant fungal phyla (**Figure 2C**). The relative abundance of *Ascomycota* was significantly more abundant in ECM and ECM.S than in the corresponding controls (*P* < 0.05).

At the class level, *Pezizomycetes* (average 30.59%), *Agaricomycetes* (15.70%), and *Sordariomycetes* (8.47%) were the dominant taxa in all the samples (**Figure 2D**). *Pezizomycetes* was significantly more abundant in ECM.S than in CK.S (*P* < 0.05). ECM harbored more *Pezizomycetes* and fewer *Agaricomycetes* and *Sordariomycetes* than CK (*P* < 0.05).

Of the 181 genera observed (**Supplementary Figure S2**), the most abundant genera were *Tuber* (with average relative abundance of 96.82% in ECM and 41.65% in ECM.S), *Acremonium*, *Schizothecium*, *Buellia*, *Staphylotrichum*, and *Phaeoacremonium* (**Figure 3B**). Not surprisingly, *Tuber* was only detected in the *T. indicum*-treated samples (ECM.S and ECM). Except *Tuber*, none of the top 25 genera had higher relative abundance in ECM than in CK. The inoculation of *T. indicum* decreased the relative abundance of the predominant genera such as *Acremonium*, *Aspergillus*, *Penicillium*, and *Claviceps* in *Q. aliena* roots (*P* < 0.05, **Supplementary Figure S3C**). In soil, the genera such as *Aspergillus*, *Phaeoacremonium*, *Pochonia*, and *Wallemia* were significantly more abundant in *T. indicum*-inoculated samples (ECM.S) than in the controls (CK.S) (*P* < 0.05, **Supplementary Figure S3D**).

### Structural Differentiation of Microbial Communities

**FIGURE 4:**
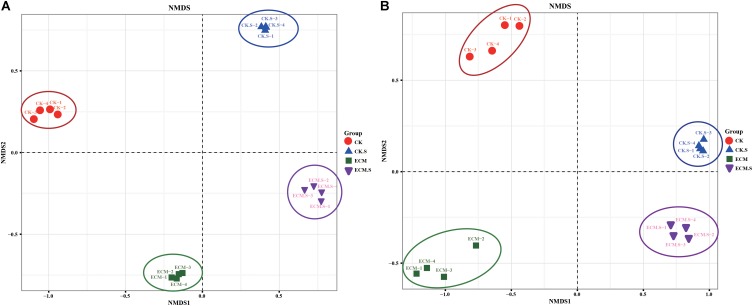
Non-metric multidimensional scaling (NMDS) analysis of bacterial **(A)** and fungal **(B)** communities in *Q. aliena* roots and the surrounding soil and with or without *T. indicum* partner. ECM, ectomycorrhizae; ECM.S, ectomycorrhizosphere soil; CK, roots of *Q. aliena* without *T. indicum* partner; CK.S, rhizosphere soil of *Q. aliena* without *T. indicum* partner.

The variations of bacterial and fungal communities among the samples were visualized using Non-metric multidimensional scaling (NMDS) analysis (**Figure 4**). The microbial (both prokaryotic and fungal) community structures were dependent on the sample type (soil versus roots) and *T. indicum*-inoculation dependent. NMDS1 separated the prokaryotic (**Figure 4A**) and fungal (**Figure 4B**) communities by the sample type, whereas NMDS2 separated the microbial communities by the *T. indicum* treatment.

### Metabolic Functions of the Bacterial Community

**FIGURE 5:**
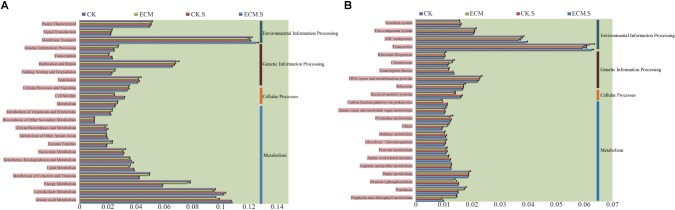
The relative abundance of the putative metabolic functions of the bacterial community in *Q. aliena* roots and the surrounding soil, predicted using PICRUSt software based on the KEGG database at the second **(A)** and third **(B)** classification level. ECM, ectomycorrhizae; ECM.S, ectomycorrhizosphere soil; CK, roots of *Q. aliena* without *T. indicum* partner; CK.S, rhizosphere soil of *Q. aliena* without *T. indicum* partner. The X axis indicates the relative abundance of the metabolic functions of the prokaryotic community. Data of the above metabolic function analysis are presented as mean ± SD (error bars), calculated from the four biological replicates of each treatment group.

The metabolic functions of the bacterial community were predicted using PICRUSt software based on the classification of the microbial metabolic functions present against the KEGG database, the COG database, and the Rfam database. Metabolism, genetic information processing, and environmental information processing were the most enriched KEGG pathways in all samples. At the second level, membrane transport, replication and repair, amino acid metabolism, carbohydrate metabolism, and energy metabolism were the most abundant predicted KEGG metabolic functions of the bacterial communities in the roots and the rhizosphere soil of *Q. aliena* (**Figure 5A**). The predicted membrane transport function was more abundant, whereas carbohydrate metabolism and replication and repair functions were less abundant in the ectomycorrhizosphere soil than in the *T. indicum*-free rhizosphere soil (*P* < 0.05). ECM samples contained more abundant amino acid metabolism and less abundant replication and repair functions than the CK samples (*P* < 0.05). At the third level, transporters and ABC transporters DNA repair and recombination proteins and purine metabolism constituted the most abundant predicted KEGG metabolic functions of the bacterial communities in the roots and the surrounding soil of *Q. aliena* (**Figure 5B**). The ECM.S samples contained more abundant transporters and ABC transporters and less abundant DNA repair and recombination and purine metabolism functions than the CK.S samples (*P* < 0.05). Transporters, DNA repair and recombination proteins and purine metabolism were less abundant in ECM samples than in the CK samples (*P* < 0.05).

**FIGURE 6:**
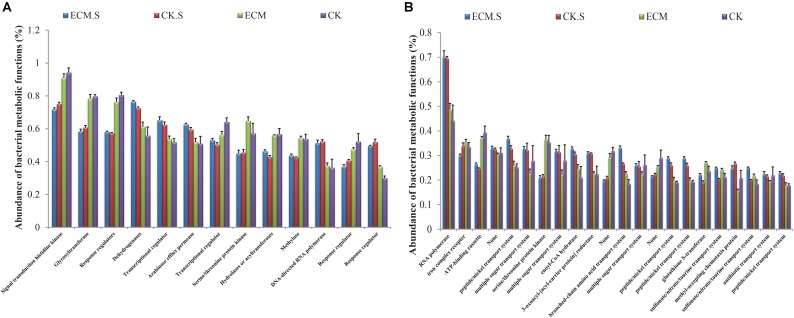
The relative abundance of putative metabolic functions of the bacterial community in *Q. aliena* roots and the surrounding soil predicted using PICRUSt software based on the COG **(A)**, and KEGG **(B)** database. ECM, ectomycorrhizae; ECM.S, ectomycorrhizosphere soil; CK, roots of *Q. aliena* without *T. indicum* partner; CK.S, rhizosphere soil of *Q. aliena* without *T. indicum* partner. Data of the above metabolic function analysis are presented as mean ± SD (error bars), calculated from the four biological replicates of each treatment group.

Signal transduction histidine kinase, glycosyltransferase, and response regulators were the most abundant metabolic functions in all the samples predicted based on the COG database (**Figure 6A**). All of these metabolic functions were more abundant in *Q. aliena* root samples than the surrounding soil samples (*P* < 0.05). RNA polymerase, iron complex receptor and ATP-binding cassette were the most abundant pathways predicted in all the samples based on the KEGG database (**Figure 6B**). The soil samples contained more abundant RNA polymerase functions and less abundant ATP-binding cassette than the root samples. The ECM sample contained more abundant RNA polymerase and iron complex receptor functions than the CK samples. ATP-binding cassette was significantly more abundant in ECM.S than in CK.S (*P* < 0.05).

### Fungal Functional Group Analysis

**FIGURE 7:**
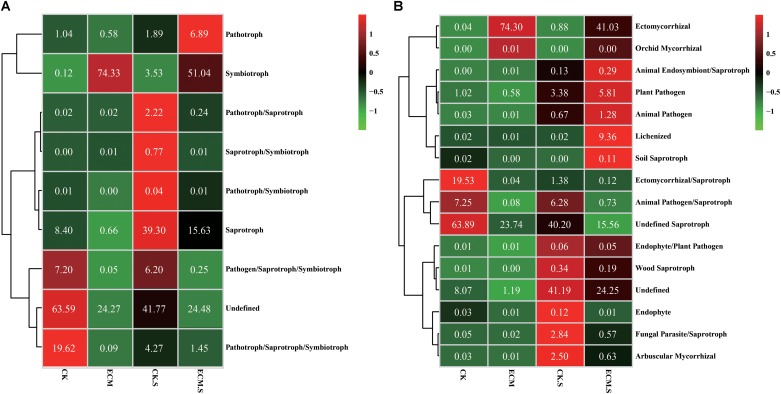
Heat-map analysis of the fungal trophic mode **(A)** and functional group (guild) **(B)** in *Q. aliena* roots and the surrounding soil, inferred by FUNGuild. ECM, ectomycorrhizae; ECM.S, ectomycorrhizosphere soil; CK, roots of *Q. aliena* without *T. indicum* partner; CK.S, rhizosphere soil of *Q. aliena* without *T. indicum* partner. The values displayed in each color block indicate the relative abundance (%) of the corresponding fungal trophic mode or the functional group.

The fungal functional group (guild) of the OTUs was inferred using FUNGuild v1.0. Regarding the trophic mode of the fungal community (**Figure 7A**), the ECM samples showed higher abundance of symbiotroph and lower abundance of pathogen/saprotroph/symbiotroph and pathotroph/saprotroph/symbiotroph modes than the CK samples (*P* < 0.05). The ECM.S samples contained more abundant pathotroph and pymbio troph and less abundant pathotroph/saprotroph, saprotroph/ symbiotroph, pathotroph/symbiotroph, saprotroph, pathogen/ saprotroph/symbiotroph, and pathotroph/saprotroph/ symbiotroph modes than the CK.S samples (*P* < 0.05).

Regarding fungal functional group (guild) (**Figure 7B**), the ECM samples contained more ectomycorrhizal and orchid mycorrhizal functional groups and less ectomycorrhizal/saprotroph, animal pathogen/saprotroph and undefined saprotroph functional groups than the CK samples (*P* < 0.05). The ECM.S samples contained more ectomycorrhizal, animal endosymbiont/saprotroph, plant pathogen, animal pathogen, lichenized, and soil saprotroph groups and less wood saprotroph, endophyte, fungal parasite/saprotroph, and arbuscular mycorrhizal groups than the CK.S samples (*P* < 0.05).

### Characteristics of Soil Properties and Correlation Analysis

Most of the soil physicochemical properties detected in this study significantly differed between ECM.S and CK.S (**Table 2**). The pH of the ectomycorrhizosphere soil varied from 8.61 to 8.64. The ectomycorrhizosphere soil (ECM.S) contained more organic matter, total nitrogen, available nitrogen, and available magnesium than the control soil (CK.S) (*P* < 0.05). The content of available phosphorus, available potassium, and available calcium was significantly lower in ECM.S than in CK.S (*P* < 0.05). This study revealed significant correlations between the measured soil properties and the microbial indices (including the alpha diversity indices and abundance of the major bacterial phyla) in soil (**Supplementary Table S1**) and in roots (**Supplementary Table S2**).

**Table 2:** Properties of the *Quercus aliena* rhizosphere and the ectomycorrhizosphere soil.

Sample	pH	OM (g/kg)	TN (g/kg)	TP (g/kg)	TK (g/kg)	AN (mg/kg)	AP (mg/kg)	AK (mg/kg)	ACa (cmol/kg)	AMg (cmol/kg)
ECM.S	8.62 ± 0.02^∗^	29.17 ± 0.25^∗^	0.94 ± 0.01^∗^	0.71 ± 0.00	23.37 ± 0.43	46.00 ± 1.73^∗^	14.83 ± 1.37^∗^	122.33 ± 0.58^∗^	51.43 ± 1.06^∗^	1.40 ± 0.10^∗^
CK.S	8.55 ± 0.01	20.90 ± 0.52	0.75 ± 0.00	0.72 ± 0.01	22.69 ± 0.55	28.00 ± 1.73	25.23 ± 0.80	642.00 ± 3.00	58.33 ± 1.51	0.90 ± 0.10


*OM, organic matter; TN, total nitrogen; TP, total phosphorus; TK, total potassium; AN, available nitrogen; AP, available phosphorus; AK, available potassium; ACa, available calcium; AMg, available magnesium. CK.S, rhizosphere soil; ECM.S, ectomycorrhizosphere soil. Each mean (±SD) was calculated from triplicates of the same treatment group. ^∗^Indicates significant differences (*P* < 0.05) between samples.*

### Metabolomic Profiling and Differentially Expressed Metabolites Between Ectomycorrhizae and the Control Roots

**FIGURE 8:**
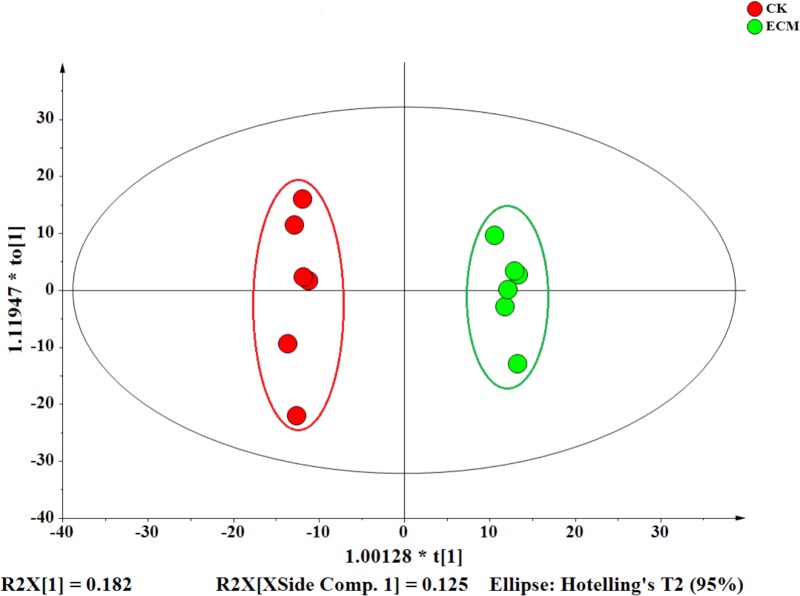
Orthogonal-partial least squares-discriminant analysis (OPLS-DA) of the metabolites in the ectomycorrhiza and control roots. The OPLS-DA score plot was generated from all of the 14704 metabolites detectedin different samples.

*Quercus aliena* root tips infected with or without *T. indicum* were sampled for LC–MS analysis. Orthogonal-partial least squares-discriminant analysis (OPLS-DA) clearly divided the 12 samples (including six ECM and six CK samples) into two groups (**Figure 8**). The first two principal components explained 30.7% of the overall variance of the metabolite profiles (18.2% for PC1 and 12.5% for PC2).

**FIGURE 9:**
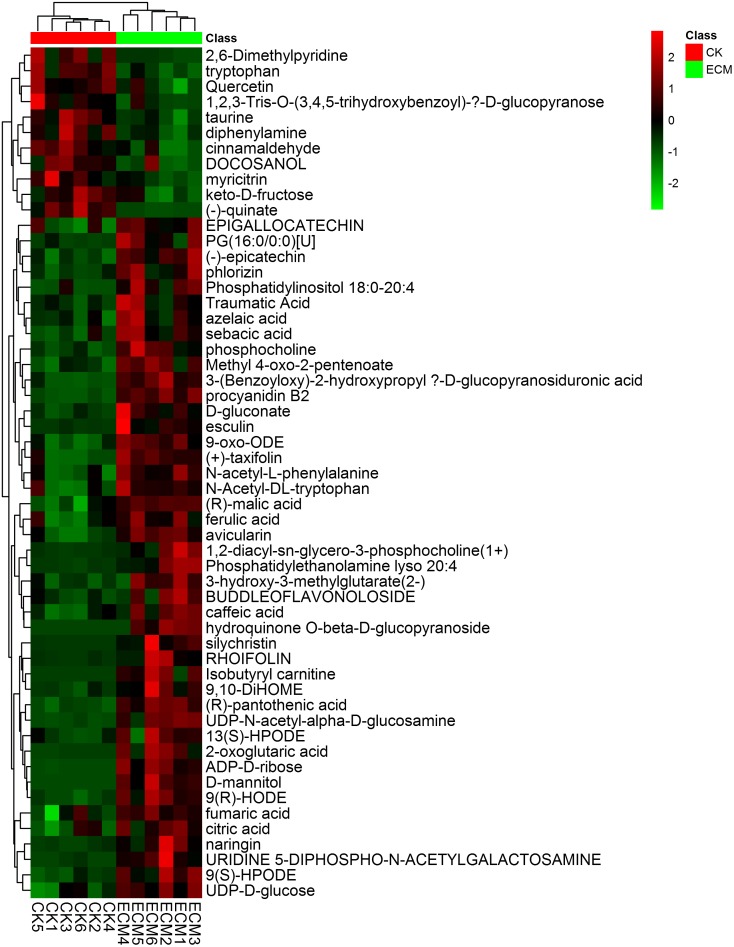
Heat-map analysis of the 55 identified metabolites that differentially expressed between the ectomycorrhizae and the control roots. ECM, ectomycorrhizae (*Q. aliena* in association with *T. indicum*); CK, roots of *Q. aliena* without *T. indicum* partner.

Independent *t*-test was performed to determine the significantly expressed metabolites of ectomycorrhizae (ECM) compared to the *T. indicum*-free control roots (CK). Of the 14704 detected metabolites, 633 metabolites showed significant differential expression patterns between ECM and CK. Fifty-five named metabolites were confirmed using National Institute of Standards and Technology (NIST) and Wiley libraries, including 44 upregulated and 11 downregulated metabolites in ECM compared with those in CK (**Figure 9**).

The metabolic pathways of the metabolites were analyzed according to the Kyoto Encyclopedia of Genes and Genomes (KEGG) database. The 55 identified metabolites that showed differentially expression patterns between ECM and CK covered 50 pathways or metabolisms, including the linoleic acid metabolism, citrate cycle (TCA cycle), alanine, aspartate, and glutamate metabolism, flavonoid biosynthesis and butanoate metabolism. Particularly, the Linoleic acid metabolism was significantly enriched in ECM with regard to CK (**Supplementary Figure S4**).

We then calculated the variations of the differentially expressed metabolites to further reveal the metabolic differentiation in *Q. aliena* root tips mycorrhized with or without *T. indicum* partner. These 55 differentially expressed metabolites included 19 organic acids, 9 amines and carbohydrates, 8 flavonoids, and 3 amino acids. The 44 metabolites that were significantly upregulated in ECM were mainly composed of organic acids and carbohydrates, including (R)-malic acid, (R)-pantothenic acid, 2-oxoglutaric acid, azelaic acid, caffeic acid, ADP-D-ribose and keto-D-fructose (**Supplementary Figure S5**). The 11 metabolites that were significantly downregulated in ECM were mainly composed of flavonoids and amino acids, including myricitrin, Quercetin, taurine, and tryptophan.

### Metabolite–Metabolite Correlation Analysis

Person correlation coefficient analysis was used to analyze the metabolite–metabolite interactions among the identified metabolites in *Q. aliena* root tips. There were 729 significant interactions (*P* < 0.05), including 503 positive and 226 negative interactions (**Supplementary Figure S6**). Notably, organic acids dominated the significant metabolite correlations, followed by carbohydrates.

## Discussion

### Truffle Affects the Microbial Diversity in Ectomycorrhizae and Ectomycorrhizosphere Soil

Because most of the microbes in nature are unculturable, the application of high throughput sequencing facilitates us to gain a comprehensive perspective on ectomycorrhizae associated microbial communities (Zhou et al., 2017). In the present study, the inoculation of *T. indicum* on *Q. aliena* roots significantly increased prokaryotic diversity and richness in both *Q. aliena* roots and the surrounding soil, in contrast with the finding that the ectomycorrhization of *T. indicum* with *P. armandii* reduces the bacterial and fungal diversity in the host roots and the surrounding soil (Li et al., 2017). Zampieri et al. (2016) have also reported that the brûlé reduces the diversity of plant and microbial species. As the environmental condition and the host plant species differ in these studies, one might suggest that truffles have different effects on the associated bacterial communities in varied environments. The diversity and richness of fungi in ectomycorrhizae and ectomycorrhizosphere soil were lower than the corresponding controls, in accordance with the previous study (Li et al., 2017). These results indicate that the effects of the ectomycorrhizal fungus on the diversity of the associated bacteria and fungi are different. Nevertheless, *T. indicum* changed the microbial community structures in the host roots and the surrounding soil, likely suggesting that the ecotomycorrhization of *T. indicum* plays important ecological roles to affect the associated microbial communities.

### Truffle Affects the Dominant Microbial Populations in Ectomycorrhizae and Ectomycorrhizosphere Soil

Our work revealed that the relative abundance of *Alphaproteobacteria* was higher in ECM than in CK, in line with previous finding that *Alphaproteobacteria* were the predominant components of truffle bacterial communities (Li et al., 2017). As *Alphaproteobacteria* are closely related to the presence of truffle mycelia, they may play an important role in the growth or ectomycorrhizal synthesis of truffles. In the present study, the bacterial genera *Pedomicrobium*, *Variibacter*, *Woodsholea*, and *Devosia* were detected more abundant in ECM or ECM.S compared with corresponding controls; the fungal genera, *Aspergillus*, *Phaeoacremonium*, *Pochonia*, and *Wallemia* were found significantly more abundant in ECM.S compared with CK.S. Previous studies have demonstrated that *T. melanosporum* ascocarps can selected specific bacterial communities from the surrounding soil to contribute to the development, maturation and even aroma of the black truffle (Vahdatzadeh et al., 2015; Deveau et al., 2016). This study, together with our previous research (Li et al., 2017), could further uncover that the truffle *T. indicum* could shape the microbial communities by selecting specific microbial groups in the surrounding environment. Interestingly, we found that truffle-colonization reduced the diversity and abundance of some fungal genera, such as *Acremonium*, *Aspergillus*, *Penicillium*, and *Claviceps*, surrounding the host tree. This finding is consistent with the results of previous studies that ectomycorrhizal fungi can enhance the resistance of host plants to pathogenic fungi by reducing the number of infected pathogenic microbial populations to plants (Jung et al., 2012; Yanan et al., 2015).

In the present study, the dominant bacterial and fungal genera (except *Tuber*) detected in the ectomycorrhizosphere soil and ectomycorrhizae were different from those revealed in the ectomycorrhizosphere soil and ectomycorrhizae of *P. armandii* mycorrhized by *T. indicum* (Li et al., 2017). The differentiation in the microbial communities is most likely caused by different host plants that provide different rhizosphere and endosphere niches (Beckers et al., 2017). The dominance of *Tuber* in the *T. indicum*-treated samples is not surprising, as the truffle mycelia has already dominated the microbial communities of ectomycorrhizosphere soil and ectomycorrhizae from the early stage of ectomycorrhizal synthesis (Li et al., 2017).

### Microbial Putative Metabolic Functions Are Altered by the Presence of Truffle

Based on the metabolic functions prediction against KEGG database, membrane transport (particularly transporters and ABC transporters) and amino acid metabolism were increased in prokaryotic communities of the surrounding soil and roots associated with *T. indicum*, with regard to the corresponding *T. indicum*-free controls. Transporters and ABC transporter proteins are all predicted to have an important role in the mycorrhizal symbiosis (Kovalchuk et al., 2015; MacLean et al., 2017; Tian et al., 2017). Our finding might therefore suggest that the activities of ectomycorrhizosphere bacteria are beneficial to mycorrhizal synthesis. Carbohydrate metabolism and replication and repair (particularly DNA repair and recombination proteins) were decreased in prokaryotic communities of the host roots and the surrounding soil in the presence of *T. indicum*. Microorganisms need to preserve genetic information to prevent the detrimental effects produced by DNA replication and the repair of damage induced by exogenous factors (Zlotorynski, 2016; Chalissery et al., 2017). Hence, this result possibly indicated that the growth of *T. indicum* could enhance the resistance of the associated bacteria to environmental stresses and thereby create an optimum environment for bacterial metabolic activities. The putative metabolic functions analysis further indicated that the colonization of truffles led to differentiation in metabolic functions of the prokaryotic community associated with the host plant. The putative metabolic functions of the microbial communities were also differentiated between roots and the surrounding soil. Based on the COG database, the dominant metabolic functions, signal transduction histidine kinase, glycosyltransferase and response regulators were more abundant in root samples than the soil samples, reflecting that the differentiation of niches could also lead to the variations of metabolic functions of the prokaryotic communities.

It is not surprising that the root and soil samples showed higher abundance of symbiotroph mode and lower abundance of Saprotroph mode in the presence of *T. indicum*, based on fungal trophic mode analysis. Regarding fungal functional group (guild), arbuscular mycorrhizal groups were less abundant in the ectomycorrhizosphere soil than in the *T. indicum*-free rhizosphere soil. The result might indicate that the growth of arbuscular mycorrhizal fungi in the ectomycorrhizosphere soil was inhibited by the presence of *T. indicum* during the early symbiotic stage. Interestingly, the orchid mycorrhizal group was found more abundant in the ectomycorrhizae than in the control roots. However, the function of the orchid mycorrhizal fungal group on the growth and ectomycorrhizal synthesis of truffle is not yet known (Leonardi et al., 2013).

### Variations in Ectomycorrhizosphere and Rhizosphere Soil Properties

In this study, the measured soil physicochemical properties, such as the pH, organic matter, total nitrogen, available nitrogen, available magnesium, available phosphorus, available potassium, and available calcium, significantly differed between the ectomycorrhizosphere soil and the *T. indicum*-free rhizosphere soil. This finding indicates that the ectomycorrhization has a feedback effect (modification) on soil properties, consistent with the previous studies (Li et al., 2017; Yang et al., 2017). The soil properties, such as the soil pH, carbonate content and available calcium content, play an important role in the production of truffles (Valverde-Asenjo et al., 2009; Garcia-Barreda et al., 2017). Therefore, the changes in soil properties may affect the growth and mycorrhizal synthesis of *T. indicum*. The ectomycorrhizosphere soil in the present study was alkalescent, which is considered suitable for the cultivation of different varieties of truffles (Garcia-Montero et al., 2008; Salerni et al., 2014). However, the ectomycorrhizosphere soil (*T. indicum* mycorrhized with *P. armandii*) was found slightly acidic in our previous study (Li et al., 2017). The contrasting results indicated that host plant species or the host-truffle interaction can affect the pH of the ectomycorrhizosphere soil. Moreover, some soil physical and chemical properties were significantly correlated with the estimated microbial diversity indices and the relative abundance of the dominant microbial populations in both ectomycorrhizae and the ectomycorrhizosphere soil. This study further confirms the existence of an interactive network among the truffle, the host plant, soil properties and the associated microbial communities as revealed by Li et al. (2017).

### Changes of Metabolic Profiles in Ectomycorrhiza and Control Roots

Orthogonal-partial least squares-discriminant analysis analysis revealed that the colonization of *T. indicum* signficantly changed the metabolic profiles in roots. We identified 55 named metabolites that showed significant differential expression patterns between the ectomycorrhizae and *Q. aliena* roots. The majority (88%) of them were significantly upregulated in the presence of *T. indicum*. These upregulated metabolites are mainly composed of organic acids and carbohydrates, such as (R)-malic acid, (R)-pantothenic acid, 2-oxoglutaric acid, azelaic acid, caffeic acid, ADP-D-ribose, and keto-D-fructose. As the main carbon sources transferred to arbuscular mycorrhizal fungi from host plants (Luginbuehl et al., 2017), organic acids were also found more abundant in the AM (Mechri et al., 2014). Mycorrhizal fungi benefit from the host plants with carbohydrates supply (Doidy et al., 2012; Ait Lahmidi et al., 2016). Therefore, our findings suggest that organic acids and carbohydrates are the two most important metabolites (carbon and energy sources) transferred between the ectomycorrhizal fungi *T. indicum* and the host plant to sustain the metabolic activities and functions in the truffle ectomycorrhization system.

In our study, the colonization of *T. indicum* decreased the content of flavonoids and amino acids (including myricitrin, quercetin, taurine, and tryptophan) detected in the host roots. Flavonoids, consisting of a group of secondary metabolites derived from the phenylpropanoid pathway, are involved in the process of regulation of arbuscular mycorrhizal colonization in the host plant roots; however, the root endosymbioses are highly complex, which is not only associated to flavonoids, but also to AM fungal genus or even species (Scervino et al., 2005; Abdel-Lateif et al., 2012). The metabolic components detected in the truffle ectomycorrhization system were highly correlated with each other. Notably, organic acids dominated the significant metabolite–metabolite interactions, followed by carbohydrates, indicating their great importance in the metabolic functioning and transmission in the truffle ectomycorrhization system. In spite of the importance of EM associations for plant vitality, previous studied failed to provide information on the molecular and physiological mechanisms of ectomycorrhizal synthesis (Mucha et al., 2014; Shah et al., 2016).

The ectomycorrhizal synthesis is crucial for efficient cultivation of truffles (Iotti et al., 2016; Marozzi et al., 2017). In the field, a variety of factors have been found to affect the microbial communities associated with truffle ectomycorrhizae, such as the host species, the age of the plantation, the surrounding environment and the management practices (Leonardi et al., 2013; Fu et al., 2016). Different experimental conditions and methods can also induce dynamic changes in microbial community structure in time and space (Benucci and Bonito, 2016). In this study, a controlled artificial ectomycorrhizal synthesis system was established with *T. indicum* and *Q. aliena* to systematically test the influence of ectomycorrhizal synthesis on microbial communities in ectomycorrhizae and the surrounding soil, as well as on the metabolic profiling of ectomycorrhizae during the symbiotic process. In conclusion, this study illustrates an interactive network constituted by *T. indiccum*, *Q. aliena*, soil and the associated microbial communities and reveals the influence of truffle colonization on the differentiation of metabolic components of ectomycorrhiza, providing insights to understand the ecological effect of truffles.

## Author Contributions

QL, WP, and XL conceived and designed the experiments. QL, LY, JZ, BZ, and XZ performed the experiments. QL, LJY, and XL wrote and revised the paper. All authors approved the final version of the manuscript.

## Conflict of Interest Statement

The authors declare that the research was conducted in the absence of any commercial or financial relationships that could be construed as a potential conflict of interest.

## Footnotes

1 http://www.kegg.jp/

2 http://www.cytoscape.org/
