# Supplementary material for: Chinese Black Truffle (Tuber indicum) Alters the Ectomycorrhizosphere and Endoectomycosphere Microbiome and Metabolic Profiles of the Host Tree Quercus aliena
Source: Front Microbiol. 2018 Sep 18;9:2202. doi: 10.3389/fmicb.2018.02202 (PMC6156548; doi:10.3389/fmicb.2018.02202)
Supplement: Supplementary file 1 [file Data_Sheet_1.DOCX]

**Table S1 Spearman correlation coefficient (rho) between soil properties and indicators of bacterial community structure.**

|  |  | pH | OM | TN | TP | TK | AN | AP | AK | ACa | AMg |
| --- | --- | --- | --- | --- | --- | --- | --- | --- | --- | --- | --- |
| Root | Observed species | 0.90* | 0.95** | 0.96** | -0.23 | 0.70 | 0.93** | -0.93** | -0.96** | -0.95** | 0.94** |
|  | Chao1 | 0.66 | 0.61 | 0.60 | 0.36 | 0.46 | 0.50 | -0.54 | -0.59 | -0.58 | 0.42 |
|  | ACE | 0.66 | 0.61 | 0.61 | 0.33 | 0.48 | 0.51 | -0.55 | -0.60 | -0.59 | 0.44 |
|  | Shannon | 0.79 | 0.79 | 0.79 | 0.27 | 0.55 | 0.69 | -0.70 | -0.77 | -0.75 | 0.62 |
|  | Simpson | 0.77 | 0.84* | 0.82* | 0.44 | 0.28 | 0.77 | -0.75 | -0.80 | -0.67 | 0.64 |
|  | Proteobacteria | 0.40 | 0.38 | 0.44 | -0.63 | 0.78 | 0.36 | -0.36 | -0.44 | -0.61 | 0.55 |
|  | Actinobacteria | 0.02 | -0.02 | 0.05 | -0.50 | 0.61 | -0.07 | 0.08 | -0.04 | -0.27 | 0.15 |
|  | Bacteroidetes | -0.57 | -0.57 | -0.62 | 0.78 | -0.83* | -0.61 | 0.60 | 0.63 | 0.78 | -0.75 |
|  | Chloroflexi | 0.97** | 0.96** | 0.96** | -0.17 | 0.77 | 0.93** | -0.95** | -0.97** | -0.97** | 0.90* |
| Soil | Observed species | 0.72 | 0.86* | 0.86* | -0.08 | 0.40 | 0.85* | -0.81 | -0.86* | -0.77 | 0.87* |
|  | Chao1 | 0.81* | 0.91* | 0.90* | -0.01 | 0.44 | 0.89* | -0.87* | -0.90* | -0.80 | 0.87* |
|  | ACE | 0.79 | 0.88* | 0.87* | 0.07 | 0.40 | 0.86* | -0.84* | -0.87* | -0.77 | 0.82* |
|  | Shannon | 0.35 | 0.59 | 0.61 | -0.32 | 0.19 | 0.62 | -0.52 | -0.60 | -0.53 | 0.75 |
|  | Simpson | 0.95** | 0.99** | 0.99** | -0.15 | 0.65 | 0.98** | 0.98** | -1.00** | -0.96** | 0.95** |
|  | Proteobacteria | -0.45 | -0.35 | -0.38 | 0.72 | -0.65 | -0.42 | 0.46 | 0.40 | 0.53 | -0.47 |
|  | Actinobacteria | -0.88* | -0.89* | -0.90* | 0.11 | -0.71 | -0.85* | 0.84* | 0.89* | 0.91* | -0.81 |
|  | Bacteroidetes | -0.79 | -0.92* | -0.91* | -0.11 | -0.38 | -0.88* | 0.85* | 0.90* | 0.79 | -0.85* |
|  | Chloroflexi | 0.90* | 0.98** | 0.97** | -0.07 | 0.48 | 0.98** | -0.97** | -0.98** | -0.87* | 0.93** |

OM, organic matter; TN, total nitrogen; TP, total phosphorus; TK, total potassium; AN, available nitrogen; AP, available phosphorus; AK, available potassium; ACa, available calcium; AMg, available magnesium.

*Significant at p < 0.05; **Significant at p < 0.01.

**Table S2 Spearman correlation coefficient (rho) between soil properties and indicators of fungal community structure.**

|  |  | pH | OM | TN | TP | TK | AN | AP | AK | ACa | AMg |
| --- | --- | --- | --- | --- | --- | --- | --- | --- | --- | --- | --- |
| Root | Observed species | -0.50 | -0.50 | -0.51 | -0.07 | -0.36 | -0.46 | 0.45 | 0.48 | 0.50 | -0.38 |
|  | Chao1 | -0.62 | -0.69 | -0.68 | -0.05 | -0.29 | -0.68 | 0.65 | 0.66 | 0.60 | -0.59 |
|  | ACE | -0.63 | -0.69 | -0.68 | -0.05 | -0.30 | -0.68 | 0.66 | 0.67 | 0.61 | -0.60 |
|  | Shannon | -0.58 | -0.64 | -0.64 | 0.23 | -0.30 | -0.69 | 0.67 | 0.64 | 0.59 | -0.65 |
|  | Simpson | -0.51 | -0.53 | -0.51 | 0.02 | -0.19 | -0.56 | 0.56 | 0.51 | 0.44 | -0.44 |
|  | Ascomycota | 0.73 | 0.83* | 0.80 | -0.04 | 0.25 | .86* | -0.84* | -0.81 | -0.68 | 0.77 |
|  | Basidiomycota | -0.66 | -0.76 | -0.74 | 0.02 | -0.17 | -0.80 | 0.78 | 0.74 | 0.60 | -0.70 |
|  | Glomeromycota | -0.65 | -0.81 | -0.77 | -0.16 | -0.02 | -0.84* | 0.80 | 0.78 | 0.56 | -0.74 |
|  | Zygomycota | -0.43 | -0.45 | -0.39 | -0.73 | 0.24 | -0.41 | 0.43 | 0.38 | 0.17 | -0.16 |
| Soil | Observed species | -0.83* | -0.96** | -0.944** | -0.03 | -0.37 | -0.95** | 0.92* | 0.94** | 0.81* | -0.90* |
|  | Chao1 | -0.75 | -0.90* | -0.89* | -0.11 | -0.24 | -0.90* | 0.85* | 0.88* | 0.72 | -0.84* |
|  | ACE | -0.75 | -0.90* | -0.88* | -0.15 | -0.22 | -0.89* | 0.85* | 0.87* | 0.71 | -0.82* |
|  | Shannon | -0.39 | -0.60 | -0.64 | 0.45 | -0.34 | -0.63 | 0.54 | 0.63 | 0.61 | -0.78 |
|  | Simpson | -0.05 | -0.23 | -0.29 | 0.52 | -0.31 | -0.25 | 0.15 | 0.27 | 0.35 | -0.47 |
|  | Ascomycota | 0.94** | 0.99** | 0.99** | -0.10 | 0.62 | 0.97** | -0.97** | -0.99** | -0.94** | 0.94** |
|  | Basidiomycota | -0.91* | -0.95** | -0.93** | -0.18 | -0.40 | -0.93** | 0.93** | 0.93** | 0.80 | -0.80 |
|  | Glomeromycota | -0.85* | -0.91* | -0.93** | 0.25 | -0.72 | -0.88* | 0.87* | 0.93** | 0.94** | -0.93** |
|  | Zygomycota | 0.61 | 0.38 | 0.36 | 0.07 | 0.55 | 0.34 | -0.45 | -0.37 | -0.45 | 0.17 |

OM, organic matter; TN, total nitrogen; TP, total phosphorus; TK, total potassium; AN, available nitrogen; AP, available phosphorus; AK, available potassium; ACa, available calcium; AMg, available magnesium.

*Significant at p < 0.05; **Significant at p < 0.01.


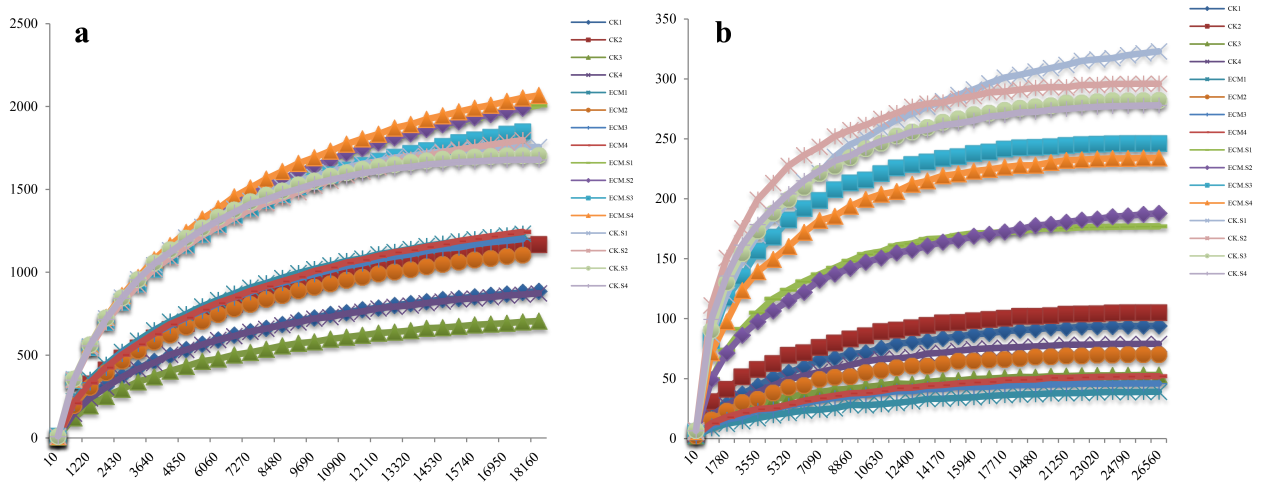


**Figure S1 Rarefaction curves for bacterial (a) and fungal (b) operational taxonomic units (OTUs) in different samples (cut-off at 97% similarity).** In the rarefaction curves, the number of OTUs increases with sequencing depths. ECM, ectomycorrhizae; ECM.S, ectomycorrhizosphere soil; CK, roots of *Quercus aliena* without *T. indicum* partner; CK.S, rhizosphere soil of *Quercus aliena* without *T. indicum* partner.


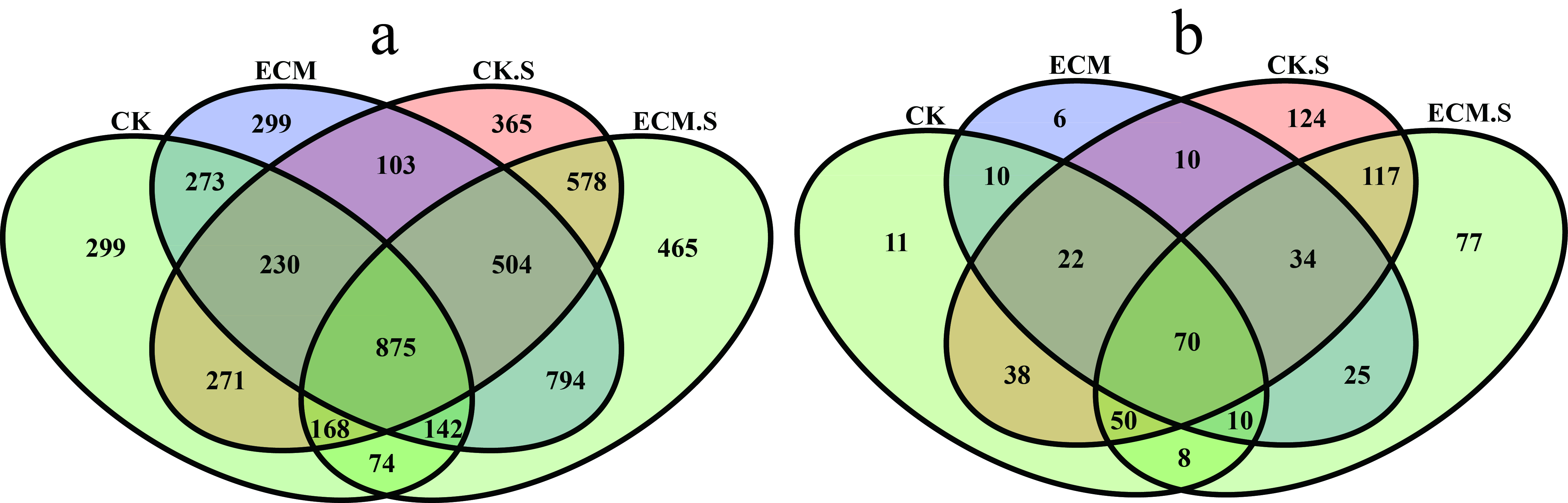


**Figure S2 The number of shared and treatment-specific bacterial (a) and fungal (b) operational taxonomic units (OTUs).** ECM, ectomycorrhizae; ECM.S, ectomycorrhizosphere soil; CK, roots of *Quercus aliena* without *T. indicum* partner; CK.S, rhizosphere soil of *Quercus aliena* without *T. indicum* partner.


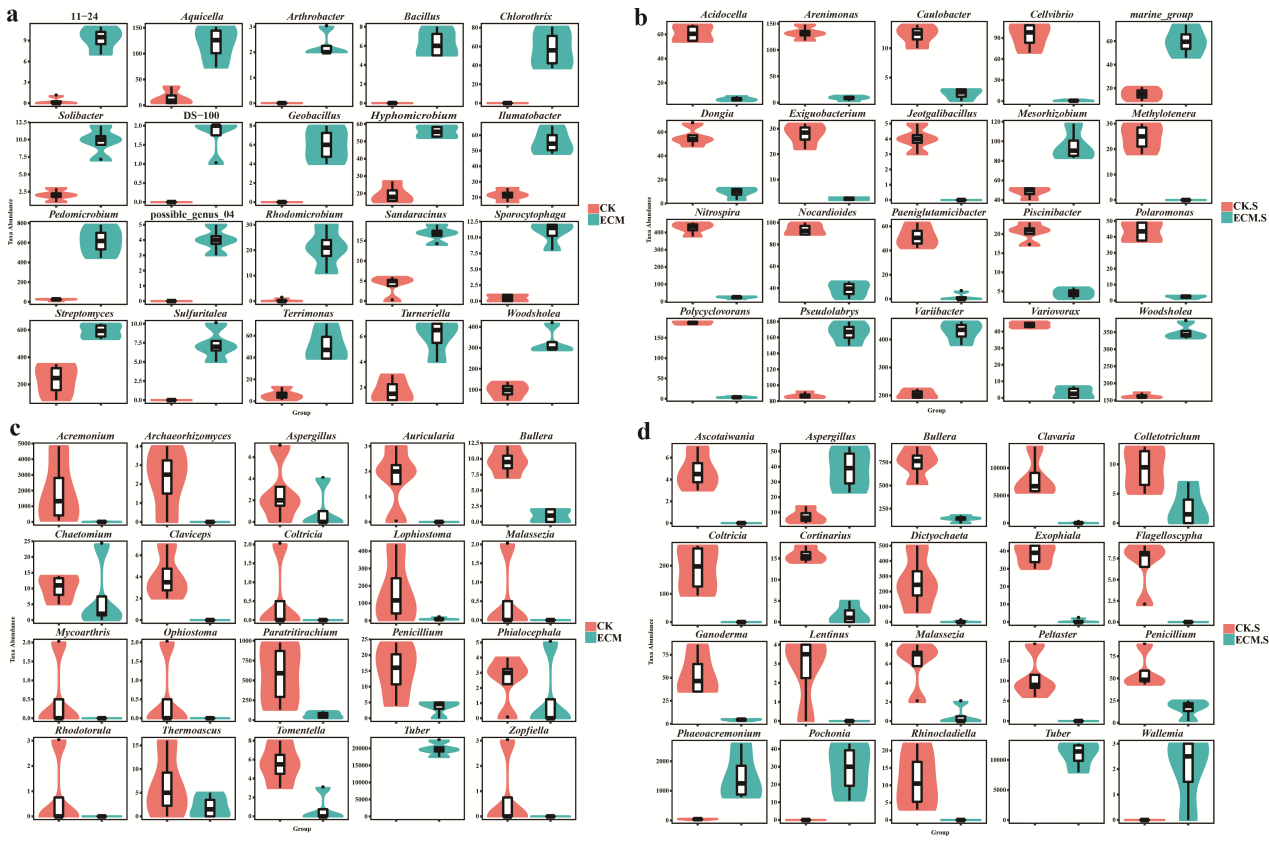


**Figure S3 Box-plot of the 25 most abundant bacterial (a and b) and fungal (c and d) genera with significant differences (p < 0.05) between different treatments.** ECM, ectomycorrhizae; ECM.S, ectomycorrhizosphere soil; CK, roots of *Quercus aliena* without *T. indicum* partner; CK.S, rhizosphere soil of *Quercus aliena* without *T. indicum* partner.


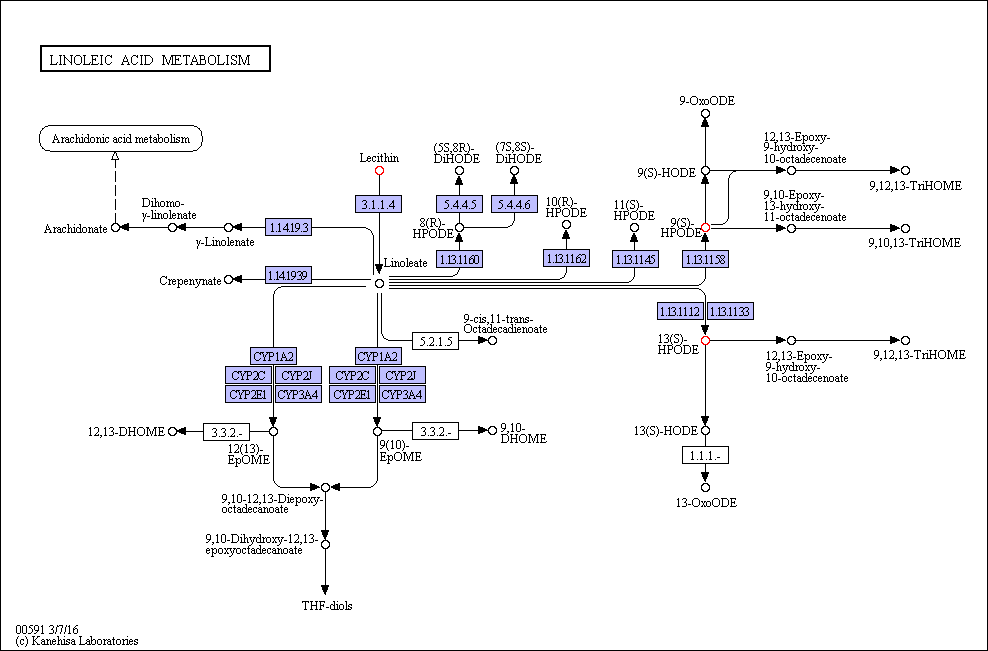


**Figure S4 The most enriched KEEG pathway (linoleic acid metabolism) of differentially expressed metabolites between the ectomycorrhiza and the control roots.** The red nodes represent the differentially expressed metabolites that participated in the displayed metabolic pathways, as revealed by this study.


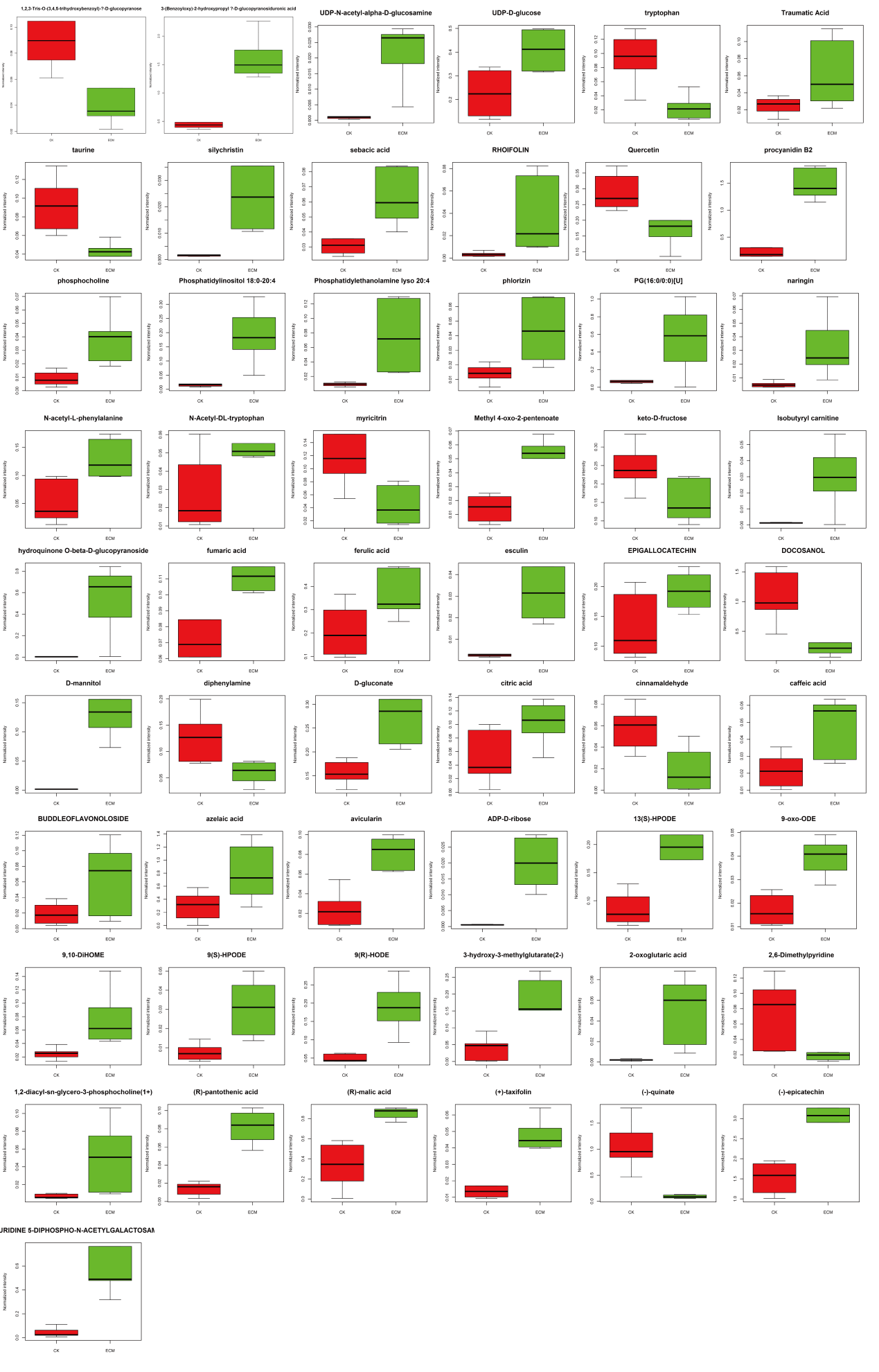


**Figure S5 Box-plot of the 55 identified metabolites differentially expressed between the ectomycorrhizae and the control roots.** ECM, ectomycorrhiza; ECM.S, ectomycorrhizosphere soil; CK, roots of *Quercus aliena* without *T. indicum* partner; CK.S, rhizosphere soil of *Quercus aliena* without *T. indicum* partner.


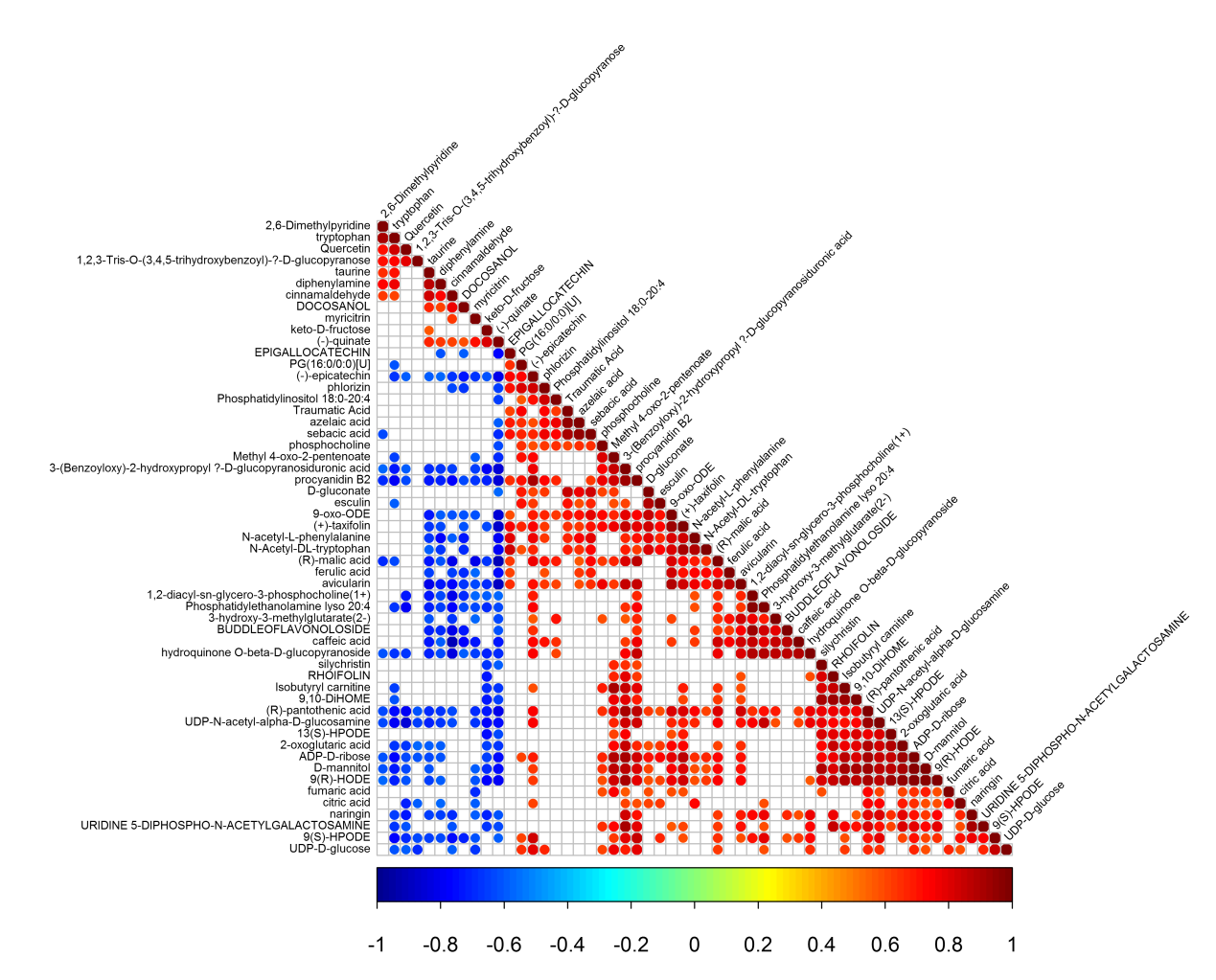


**Figure S6 Metabolite-metabolite correlation analysis.** Positive correlations are shown in red and negative correlations in blue.
